# Dietary saturated fat and docosahexaenoic acid differentially effect cardiac mitochondrial phospholipid fatty acyl composition and Ca^2+^ uptake, without altering permeability transition or left ventricular function

**DOI:** 10.1002/phy2.9

**Published:** 2013-06-12

**Authors:** Kelly A O'Connell, Erinne R Dabkowski, Tatiana de Fatima Galvao, Wenhong Xu, Caroline Daneault, Christine de Rosiers, William C Stanley

**Affiliations:** 1 Division of Cardiology, Department of Medicine, University of MarylandBaltimore, Maryland; 2 Intensive Care Unit, Hospital Israelita Albert EinsteinSao Paulo, Brazil; 3 Department of Nutrition and Montreal Heart Institute, Université de MontréalMontreal, Quebec, Canada; 4 Discipline of Physiology, University of SydneySydney, Australia

**Keywords:** Cardiovascular, mitochondria, n3-polyunsaturated fatty acids, nutrition, phospholipid, saturated fatty acids

## Abstract

High saturated fat diets improve cardiac function and survival in rodent models of heart failure, which may be mediated by changes in mitochondrial function. Dietary supplementation with the n3-polyunsaturated fatty acid docosahexaenoic acid (DHA, 22:6n3) is also beneficial in heart failure and can affect mitochondrial function. Saturated fatty acids and DHA likely have opposing effects on mitochondrial phospholipid fatty acyl side chain composition and mitochondrial membrane function, though a direct comparison has not been previously reported. We fed healthy adult rats a standard low-fat diet (11% of energy intake from fat), a low-fat diet supplemented with DHA (2.3% of energy intake) or a high-fat diet comprised of long chain saturated fatty acids (45% fat) for 6 weeks. There were no differences among the three diets in cardiac mass or function, mitochondrial respiration, or Ca^2+^-induced mitochondrial permeability transition. On the other hand, there were dramatic differences in mitochondrial phospholipid fatty acyl side chains. Dietary supplementation with DHA increased DHA from 7% to ∼25% of total phospholipid fatty acids in mitochondrial membranes, and caused a proportional depletion of arachidonic acid (20:4n6). The saturated fat diet increased saturated fat and DHA in mitochondria and decreased linoleate (18:2n6), which corresponded to a decrease in Ca^2+^ uptake by isolated mitochondria compared to the other diet groups. In conclusion, despite dramatic changes in mitochondrial phospholipid fatty acyl side chain composition by both the DHA and high saturated fat diets, there were no effects on mitochondrial respiration, permeability transition, or cardiac function.

## Introduction

There is growing evidence that the amount and type of dietary lipid affects cardiac function and survival in rodent models of heart failure, which may be partially mediated by changes in mitochondrial function (Stanley et al. 2012). High intake of long-chain saturated fat has either neutral or adverse effects on the heart in the setting of pressure overload left ventricular hypertrophy (Okere et al. 2006; Duda et al. 2008; Chess et al. 2009). In a rat model of coronary artery ligation-induced heart failure, a high saturated fat diet increases the activity of fatty acid oxidation enzymes and state 3 respiration in isolated mitochondria (Rennison et al. 2007, 2008). More recently, we have found that high intake of a mixture of long-chain saturated and monounsaturated fat prolonged survival in cardiomyopathic hamsters compared to a low-fat/high carbohydrate diet and a diet high in polyunsaturated fat (n6 and n3-polyunsaturated fatty acids [PUFAs]) (Galvao et al. 2012). On the other hand, studies with a low-fat diet supplemented with relatively low levels of the marine n3-PUFA docosahexaenoic acid (DHA) found that DHA readily incorporates into total cardiac and mitochondrial membranes (Duda et al. 2009; O'shea et al. 2009; Khairallah et al. 2010a,b, 2012; Galvao et al. 2013), and affects mitochondrial function by delaying Ca^2+^-induced mitochondrial permeability transition (MPT) (O'shea et al. 2009; Khairallah et al. 2010a,b, 2012; Galvao et al. 2013). MPT is a catastrophic event that permeabilizes the inner mitochondrial membrane, depolarizing the mitochondria and promoting apoptosis and myocardial injury.

The mechanism of the delay in Ca^2+^-induced MPT with dietary DHA supplementation is not clear. We initially thought that it was due to depletion of the n6-PUFA arachidonic acid (20:4n6) from cardiac membranes resulting in less triggering of MPT through a phospholipase A_2_ dependent mechanism (Kinsey et al. 2007; Khairallah et al. 2010b; Moon et al. 2012). We found, however, that when we elevated arachidonic acid in mitochondrial membranes with dietary arachidonic acid supplementation there was greater resistance to Ca^2+^-induced MPT. This suggests that the resistance to MPT may be due to changes in membrane viscosity subsequent to incorporation of very long-chain PUFA. If this is the case, then a high saturated fat diet should decrease membrane fatty acyl saturation and increase viscosity, resulting in greater susceptibility to MPT. In addition, the fatty acyl composition of mitochondrial membranes may affect multiple functions of mitochondria, including the rate of uptake of extramitochondrial Ca^2+^. Previous studies have not assessed the effects of increasing DHA or saturated fatty acids in mitochondria membrane phospholipids on membrane viscosity or mitochondrial Ca^2+^ uptake. Furthermore, the effect of a high saturated fat diet on cardiac mitochondrial phospholipid fatty acyl side chain composition or MPT have not been reported.

Thus, the goal of the present investigation was to compare the effects of a standard low-fat diet to a high saturated fat diet or a low-fat diet supplemented with DHA on cardiac and mitochondrial function in normal healthy rats. Animals were fed for 6 weeks, which is sufficient duration to remodel cardiac phospholipid fatty acid composition (Brochot et al. 2009). We hypothesized that DHA would be beneficial on mitochondrial function (assessed by respiration and calcium uptake) and that high saturated fat would decrease cardiac and mitochondrial function. We also expected that high DHA would increase DHA in mitochondrial membranes and a high saturated fat would increase the amount of palmitate and stearate and decrease monounsaturated fatty acids in membranes. Finally, we hypothesized that both diets would not affect cardiac function or mass. Our results show significant changes in mitochondrial phospholipid acyl side chain composition in subsarcolemmal mitochondria (SSM) and interfibrillar mitochondria (IFM) with both the DHA low-fat and the high saturated fat diets which affected mitochondrial Ca^2+^ uptake, but with no effect on mitochondrial respiration or susceptibility to MPT.

## Methods

### Experimental design

The animal protocol was conducted in accordance with the Guidelines for the Care and Use of Laboratory Animals (National Institutes of Health Publication No. 85-23) and was approved by the University of Maryland Institutional Animal Care and Use Committee. Healthy, male Sprague-Dawley rats (Harlan, Indianapolis, IN) (∼320 g) were assigned to either a standard low-fat diet (*n* = 28), a low-fat diet supplemented with DHA (*n* = 30), or a high-fat diet comprised mainly of saturated fat (*n* = 30). The animals were maintained on a 12-h light/dark cycle, and procedures and tissue harvest were performed within 4 h of the start of the light cycle. Echocardiography was performed after 6 weeks of treatment, and animals were then euthanized.

### Diets

All diets were custom manufactured by Research Diets Inc. (New Brunswick, NJ) and contained 20% of total energy content as protein from (casein + l-cystine) (Table 1). The standard control low-fat diet contained 11% of total energy from fat (4% from unsalted butter, 5% from lard, and 2% from soybean oil) and 69% carbohydrate (56% corn starch and 12% maltodextrin). The DHA diet was the same as the standard control low-fat diet except it contained 2.3% of energy intake as DHA (DHA ethyl ester, 90% pure, from KD Pharma, Bexbach, Germany), 3% unsalted butter, 4% lard, 2% soybean oil. This level of DHA is approximately equivalent to 5 g/day in humans, assuming an energy intake of 2000 kcals/day and 9 kcals/g of DHA. The high saturated fat diet contained 45% of total energy from fat (44% unsalted butter and 2% soybean oil) and 35% carbohydrate (23% corn starch and 12% maltodextrin). The fatty acid composition of the diets was analyzed by gas chromatography – mass spectroscopy as described below, and is given in Table 1. The high saturated fat diet derived 31% of total energy from saturated fat, compared to 5% and 4% for the standard low-fat and DHA diets, respectively.

**Table 1:** Analysis of fatty acids in rodent chow (expressed as a % of total fatty acids)

	Standard low-fat diet	DHA low-fat diet	High saturated fat diet
% kcal			
Protein	20	20	20
Carbohydrate	69	68	35
Fat	11	11	45
Fatty acid			
C14:0	0.1	0.1	5.1
C16:0	2.5	1.9	17.8
C16:1	0.2	0.2	0.9
C18:0	2.7	2.1	8.2
C18:1n9	3.1	2.5	11.0
C18:1n7	0.3	0.2	0.5
C18:2n6	1.6	1.4	2.2
C18:3n6	–	–	–
C18:3n3	0.1	0.1	0.1
C20:3n6	–	–	0.1
C20:3n9	–	–	–
C20:4	0.4	0.3	0.3
C20:5n3	–	0.2	0.1
C22:5n3	–	0.1	–
C22:6n3	–	2.0	–
% Saturated	5.2	4.1	31.0
%MUFA	3.6	2.9	12.4
%n6 PUFA	2.0	1.8	2.7
%n3 PUFA	0.1	2.3	0.2

DHA, docosahexaenoic acid; MUFA, monounsaturated fatty acid; PUFA, polyunsaturated fatty acid.

### Echocardiography

Left ventricular (LV) function was assessed using echocardiography (model Vevo 770, VisualSonics, Inc., Toronto, Canada) as previously described in detail (Duda et al. 2009). Briefly, rats were anesthetized using 2.5% isoflurane administered by nose cone, placed on a heated platform and two-dimensional long- and short-axis images as well as guided M-mode were acquired and analyzed using software resident on the machine. Absolute wall thickness was calculated as anterior wall thickness + posterior wall thickness at end diastole, and relative wall thickness as absolute wall thickness divided by end diastolic diameter.

### Tissue harvest

Rats were anesthetized with 5.0% isoflurane, and a thoracotomy was rapidly performed to expose the heart. Blood was drawn by cardiac puncture and the heart was immediately harvested. The left ventricle was dissected, weighed, and immediately used for mitochondrial isolation. The epididymal and retroperitoneal fat pad were also dissected and weighed.

### Mitochondrial isolation

In myocardium, mitochondria are located in two spatially distinct subpopulations: IFM located between the myofibrils, and SSM found in the outer region of the cell. Functional and structural differences between IFM and SSM have been described (Palmer et al. 1985, 1986; Riva et al. 2005; Asemu et al. 2013), thus it is important to separately assess the two populations. SSM and IFM were isolated according to the method of Palmer et al. (1977) with minor modifications (O'shea et al. 2009). Briefly, freshly harvested LV tissue was minced and homogenized in 1:10 ice cold modified Chappel-Perry buffer (100 mmol/L KCl, 50 mmol/L 4-Morpholinepropanesulfonic acid (MOPS), 5 mM MgSO_4_, 1 mmol/L Ethylene glycol-bis (2-aminoethylether)-N,N,N',N'-tetraacetic acid (EGTA), 1 mmol/L ATP, 0.2 mg/mL bovine serum albumin [BSA]). Homogenates were centrifuged at 500*g* to yield SSM, and IFM were extracted with tryptic digestion on ice (5 mg/g wet weight) for 10 min, along with further purification through a series of differential centrifugation spins. Mitochondrial protein was assessed by the Lowry method using a BSA standard curve.

### Mitochondrial respiration

Mitochondrial oxygen consumption was measured in SSM and IFM using a Clark-type electrode as described previously (O'shea et al. 2009). Isolated mitochondria (0.5 mg mitochondrial protein/mL) were respired in the same calcium-free buffer described above but containing 1 mg/mL BSA and no ATP. States 3 and 4 respiration were measured utilizing glutamate + malate (10 and 5 mmol/L), palmitoylcarnitine (40 μmol/L), and succinate (20 mmol/L) in combination with rotenone (7.5 μmol/L). The respiratory control ratio (RCR) was calculated as the ratio of State 3/State 4 respiration.

### Mitochondrial Ca^2+^ handling

The ability of isolated SSM and IFM to take up Ca^2+^ was assessed as previously described (Papanicolaou et al. 2012). In short, Ca^2+^ uptake was measured using the equivalent of 1.5 mg mitochondrial protein resuspended in 1.5 mL of Ca^2+^-free buffer containing 100 mmol/L KCl, 50 mmol/L MOPS, 5 mmol/L KH_2_PO_4_, 5 μmol/L EGTA, 1 mmol/L MgCl_2_, 5 mmol/L glutamate, and 5 mmol/L malate at 37°C. The membrane impermeable Ca^2+^ indicator, Ca^2+^ green-5N (Invitrogen, Carlsbad, CA), with an excitation and emission of 488 and 530 nm, was added to measure extramitochondrial Ca^2+^ fluorescence. Ca^2+^ uptake was taken from the fall in extramitochondrial Ca^2+^ following a bolus injection of 3 μL of 15 mmol/L Ca^2+^ (30 nmol Ca^2+^/mg mitochondrial protein).

### Ca^2+^-induced mitochondrial swelling

The change in absorbance at 540 nm, an established measure of Ca^2+^-induced mitochondrial swelling and permeability transition, was monitored at 37°C in isolated cardiac mitochondria using a 96 well spectrophotometic plate reader as described previously (Khairallah et al. 2012). Briefly, 50 μg of mitochondrial protein was resuspended in 200 μL of Ca^2+^-free buffer (described above), was monitored at 540 nm for 2 min to obtain a baseline, and followed by addition of a bolus of 100 nmols Ca^2+^/mg mitochondrial protein. The absorbance was recorded for 20 min. A parallel time control group without addition of a Ca^2+^ bolus was used to establish stability.

### Membrane microviscosity

Mitochondrial membrane microviscosity was assessed using fluorescence polarization of the membrane bound dyes, 1,6-diphenyl-1,3,5-hexatriene (DPH, Invitrogen) with an excitation and emission wave lengths of 360 and 430 nm and 1-(4-trimethylammoniumphenyl)-6-phenyl-1,3,5-hexatriene *p*-toluenesulfonate (TMA-DPH, Invitrogen) with an excitation and emission of 350 and 420 nm, respectively. Briefly, 200 μg mitochondrial protein was incubated in 3 mL of a Ca^2+^-free buffer (described above) with either 10 μmol/L DPH or 5 μmol/L TMA-DPH at 37°C for 30 min. Samples were vortexed and 1 mL of sample was read in a cuvette using single point polarization at 650 V, high sensitivity. The band pass was set at 4 for excitation and 8 for emission. Samples were run in duplicate and anisotropy values obtained are inversely related to membrane fluidity.

### Phospholipid fatty acyl analysis

Mitochondrial phospholipid fatty acyl composition was analyzed by gas chromatography coupled with mass spectrometry according to a modification of the transesterification method as previously described (Gelinas et al. 2011; Khairallah et al. 2012).

### Plasma analysis

Free fatty acids and triglycerides were measured in plasma by spectrophotometic enzymatic assays (Wako Chemicals, Richmond, VA).

### Statistical analyses

Differences among the three dietary treatments were assessed using a one-way analysis of variance (ANOVA), either parametric or on ranks depending if normally distributed. A Bonferroni or Dunn's post hoc test was used to compare groups, for parametric and nonparametric analysis, respectively. A two-way repeated measures ANOVA with a Bonferroni post hoc test was performed on the calcium uptake experiments. SSM and IFM were not compared. Data are presented as mean values ± SEM. A difference of *P* < 0.05 was considered significant.

## Results

### Body mass and cardiac function

Initial body mass was similar among groups, and there was significant weight gain in all groups over the 6 weeks of treatment. The high saturated fat diet group had a 42% greater gain in body mass compared to the standard low-fat diet and DHA groups (*P* < 0.05) (Table 2). This corresponded with significantly greater epididymal and retroperitoneal fat pad masses. There were no differences in absolute LV mass or LV mass when normalized to tibia length. When LV mass or biventricular mass (LV + right ventricle) were normalized to body mass values were lower in the high saturated fat diet group due to the higher body mass (Table 2). No changes were seen in LV chamber diameter or volume at end systole or diastole, in absolute or relative wall thickness, or in ejection fraction (Table 2). Taken together, there was no evidence of cardiac hypertrophy or gross cardiac dysfunction with short term diet-induced obesity with the high saturated fat diet.

**Table 2:** Physiological parameters and echocardiography data

	Standard low-fat diet	DHA low-fat diet	High saturated fat diet
*N*	18	20	19
Initial body mass (g)	318 ± 7	323 ± 4	323 ± 5
Final body mass (g)	488 ± 10	494 ± 8	554 ± 11^1^^,^^2^
Change in body mass (g)	170 ± 9	171 ± 6	242 ± 15^1^^,^^2^
LV mass (g)	0.98 ± 0.02	0.97 ± 0.02	1.02 ± 0.02
LV/tibia length (mg/mm)	23.85 ± 0.90	22.93 ± 0.51	24.52 ± 0.68
LV+RV/body weight (mg/g)	2.45 ± 0.03	2.39 ± 0.03	2.24 ± 0.03^1^^,^^2^
LV/body weight (mg/g)	2.01 ± 0.03	1.96 ± 0.03	1.85 ± 0.02^1^^,^^2^
Epididymal fat mass (g)	9.24 ± 0.85	7.27 ± 0.42	14.12 ± 1.00^1^^,^^2^
Retroperitoneal fat mass (g)	9.66 ± 1.23	7.79 ± 0.48	14.08 ± 1.02^1^^,^^2^
Echocardiography data			
EDD (mm)	7.5 ± 0.3	7.3 ± 0.2	7.2 ± 0.2
ESD (mm)	3.7 ± 0.2	3.7 ± 0.2	3.6 ± 0.2
End diastolic volume (mL)	0.47 ± 0.05	0.43 ± 0.03	0.41 ± 0.04
End systolic volume (mL)	0.06 ± 0.01	0.06 ± 0.01	0.06 ± 0.01
EF (%)	88.1 ± 1.2	86.3 ± 1.7	86.9 ± 1.5
Absolute wall thickness (mm)	4.5 ± 0.2	4.4 ± 0.2	4.3 ± 0.2
Relative wall thickness (mm)	0.6 ± 0.1	0.6 ± 0.0	0.6 ± 0.0

DHA, docosahexaenoic acid; LV, left ventricle; RV, right ventricle; EDD, end diastolic diameter; ESD, end systolic diameter; EF, ejection fraction.

1 *P* < 0.05 versus standard low-fat diet.

2 *P* < 0.05 versus DHA low-fat diet.

### Plasma lipids

**Figure 1:**
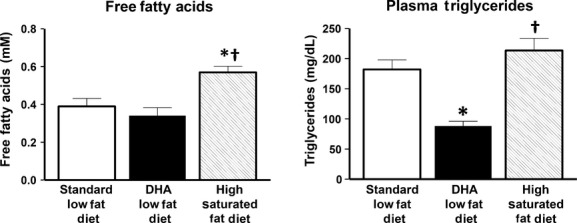
Circulating free fatty acids and plasma triglycerides. *P < 0.05 versus standard low-fat diet, ^†^*P* < 0.05 versus DHA low-fat diet. The sample size was 18, 20, and 19 for standard low fat, DHA, and high saturated fat, respectively.

As expected, DHA significantly decreased plasma triglycerides compared to the standard low-fat diet and high saturated fat diet (Fig. 1). Plasma free fatty acids were increased in animals fed the high saturated fat diet compared to the standard low-fat and DHA diets.

### Mitochondrial phospholipid composition

**Figure 2:**
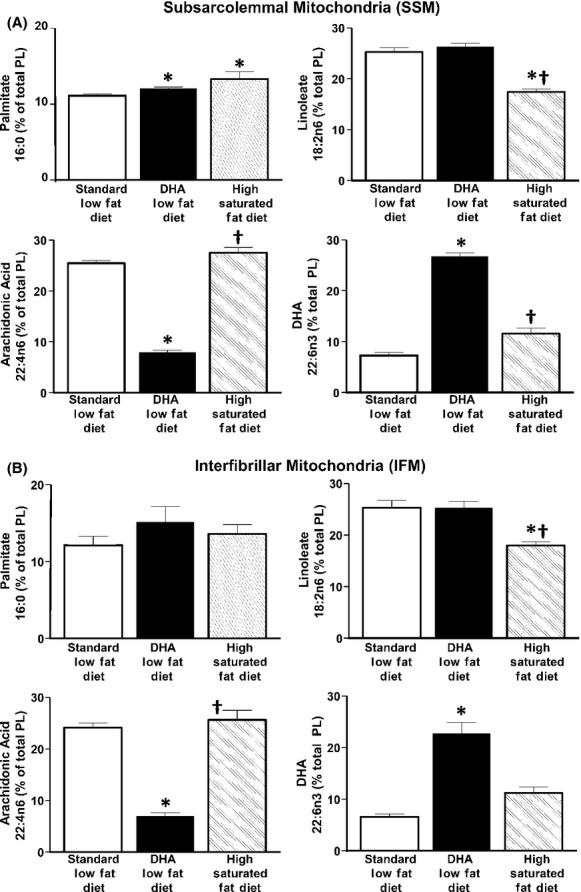
(A) Mitochondrial phospholipid analysis of palmitate (16:0), linoleate (18:2n6), arachidonic acid (20:4n6), and DHA (22:6n3) in isolated SSM. *P < 0.05 versus standard low-fat diet, ^†^*P* < 0.05 versus DHA low-fat diet. The sample size was 9, 10, and 10 for standard low fat, DHA, and high saturated fat, respectively. (B) Mitochondrial phospholipid analysis of palmitate (16:0), linoleate (18:2n6), arachidonic acid (20:4n6), and DHA (22:6n3) in isolated IFM. *P < 0.05 versus standard low-fat diet, ^†^*P* < 0.05 versus DHA low-fat diet. The sample size was 9, 10, and 8 for standard low fat, DHA, and high saturated fat, respectively.

Analysis of the fatty acyl composition of mitochondrial phospholipids revealed dramatic differences among the three diet groups. Within SSM, the DHA diet increased DHA and proportionally decreased the n6-PUFA arachidonic acid (Fig. 2A, Table 3). The high DHA diet also increased the saturated fatty acids palmitate (16:0) and stearate (18:0) while decreasing the monounsaturated and *trans*-monounsaturated fatty acids oleate (18:1n9) and vaccenic acid (18:1n7), respectively. Thus, supplementation with DHA lowered total monounsaturated fatty acids and n6-PUFA compared to the standard low-fat diet (Table 3).

**Table 3:** Mitochondrial phospholipid fatty acyl group composition expressed as a percent of total

	Standard low-fat diet	DHA low-fat diet	High saturated fat diet
SSM			
Fatty acid	*n* = 9	*n* = 10	*n* = 10
Palmitoleic acid (16:1n7)	0.4 ± 0.0	0.3 ± 0.0	0.2 ± 0.0^1^^,^^2^
Stearic acid (18:0)	23.8 ± 0.5	21.7 ± 0.3^1^	22.7 ± 1.1
Oleic acid (18:1n9)	3.3 ± 0.1	2.7 ± 0.1^1^	4.0 ± 0.3^2^
Vaccenic acid (18:1n7)	2.9 ± 0.1	1.9 ± 0.0^1^	1.9 ± 0.1^1^
α-Linolenic acid (18:3n3)	0.1 ± 0.0	0.0 ± 0.0^1^	0.1 ± 0.0^2^
Eicosapentaenoic acid (20:5n3)	0.0 ± 0.0	0.1 ± 0.0^1^	0.0 ± 0.0^2^
Docosapentaenoic acid (22:5n3)	0.2 ± 0.0	0.1 ± 0.0^1^	0.8 ± 0.2^1^^,^^2^
Σ Saturated fatty acids	35.0 ± 0.6	33.8 ± 0.4	36.1 ± 1.3
Σ Monounsaturated fatty acids	6.6 ± 0.2	4.8 ± 0.1^1^	6.1 ± 0.4^2^
Σ n3-PUFA	7.5 ± 0.7	25.0 ± 0.8^1^	12.5 ± 1.4^2^
Σ n6-PUFA	50.8 ± 0.7	34.3 ± 0.5^1^	45.0 ± 1.6^2^
IFM			
Fatty acid	*n* = 9	*n* = 10	*n* = 8
Palmitoleic acid (16:1n7)	0.4 ± 0.0	0.3 ± 0.0	0.2 ± 0.0^1^
Stearic acid (18:0)	24.8 ± 1.2	24.4 ± 1.4	24.3 ± 0.9
Oleic acid (18:1n9)	3.5 ± 0.2	2.8 ± 0.2	3.9 ± 0.4^2^
Vaccenic acid (18:1n7)	2.8 ± 0.1	1.8 ± 0.1^1^	1.9 ± 0.1^1^
α-Linolenic acid (18:3n3)	0.1 ± 0.0	0.0 ± 0.0	0.1 ± 0.0^2^
Eicosapentaenoic acid (20:5n3)	0.0 ± 0.0	0.1 ± 0.0^1^	0.0 ± 0.0
Docosapentaenoic acid (22:5n3)	0.2 ± 0.0	0.1 ± 0.0^1^	0.8 ± 0.2^2^
Σ Saturated fatty acids	36.9 ± 2.4	39.5 ± 3.5	37.9 ± 1.9
Σ Monounsaturated fatty acids	6.7 ± 0.3	5.0 ± 0.3^1^	6.0 ± 0.5
Σ n3-PUFA	6.8 ± 0.6	23.2 ± 2.2^1^	12.1 ± 1.4
Σ n6-PUFA	49.4 ± 2.3	32.3 ± 1.7^1^	43.6 ± 2.4

DHA, docosahexaenoic acid; PUFA, polyunsaturated fatty acid; SSM, subsarcolemmal mitochondria; IFM, interfibrillar mitochondria.

1 *P* < 0.05 versus standard low-fat diet.

2 *P* < 0.05 versus DHA diet.

In SSM, the high saturated fat caused modest but significant increases in palmitate and oleate compared to the standard low-fat diet (Table 3, Fig. 2A). The high saturated fat diet decreased the n6-PUFA linoleate (18:2n6) compared to both the standard and DHA diets, but had no effect on arachidonic acid compared to the standard diet. The high saturated fat diet also raised levels of docosapentaenoic acid (DPA, 22:5n3) and DHA, but not EPA, consistent with our previous measurements in whole myocardial extracts from rats fed a high saturated diet (Table 3, Fig. 2A) (Shah et al. 2009).

In IFM, the diet-induced differences in phospholipid fatty acyl side chains were generally similar to those seen in SSM (Fig. 2B, Table 3). Again, the main effect of the DHA diet was to increase DHA in membrane phospholipids in exchange for a proportional decrease in arachidonic acid. The DHA diet significantly increased EPA and decreased both palmitoleic and vaccenic acid compared with the standard low-fat diet. Oleate was increased relative to the DHA diet.

### Mitochondrial yield and respiration

The yield of the two mitochondrial subpopulations, SSM and IFM, and total mitochondrial yield were not different among the three diets (Table 4). Extensive assessment of mitochondrial respiration with various substrates found no differences in state 3 or state 4 respiration or the RCR (Table 4).

**Table 4:** Mitochondrial yield, respiration, and RCR for all substrates

	Standard low-fat diet (*n* = 20)	DHA low-fat diet (*n* = 20)	High saturated fat diet (*n* = 20)
LV Mito yield (mg protein/g wet wt)			
SSM	17.2 ± 1.3	17.3 ± 0.7	17.6 ± 0.8
IFM	11.6 ± 0.8	11.1 ± 0.5	10.7 ± 0.6
Total	28.9 ± 1.8	28.2 ± 1.0	28.3 ± 1.0
Glutamate + Malate	*n* = 9	*n* = 10	*n* = 9
SSM			
State 3	107.9 ± 9.1	105.4 ± 6.1	119.1 ± 14.4
State 4	33.0 ± 2.9	35.8 ± 3.3	31.1 ± 3.0
RCR	3.3 ± 0.1	3.1 ± 0.3	3.9 ± 0.3
IFM			
State 3	145.6 ± 13.3	129.9 ± 6.7	140.2 ± 14.2
State 4	44.4 ± 8.3	43.1 ± 4.0	36.5 ± 4.0
RCR	3.6 ± 0.2	3.2 ± 0.3	4.0 ± 0.7
Palmitoylcarnitine			
SSM			
State 3	222.2 ± 31.5	207.1 ± 20.9	224.6 ± 14.6
State 4	61.6 ± 14.8	47.3 ± 2.7	50.3 ± 4.0
RCR	4.0 ± 0.4	4.4 ± 0.3	4.6 ± 0.4
IFM			
State 3	259.4 ± 21.9	263.9 ± 19.2	256.3 ± 15.8
State 4	57.6 ± 5.8	62.5 ± 2.5	58.2 ± 4.2
RCR	4.6 ± 0.2	4.2 ± 0.3	4.6 ± 0.4
Succinate + Rotenone			
SSM			
State 3	273.1 ± 9.7	272.7 ± 10.3	262.3 ± 7.3
State 4	89.2 ± 7.4	89.8 ± 5.7	87.1 ± 6.3
RCR	3.2 ± 0.4	3.1 ± 0.2	3.1 ± 0.2
IFM			
State 3	363.3 ± 26.9	331.1 ± 11.9	338.8 ± 15.3
State 4	111.9 ± 9.7	104.6 ± 7.9	109.3 ± 5.9
RCR	3.3 ± 0.4	3.3 ± 0.3	3.1 ± 0.1

States 3 and 4 are expressed in *n*_atoms_ O·mg^−1^·min^−1^. RCR, respiratory control ratio (is the ratio of State 3/State 4); DHA, docosahexaenoic acid; SSM, subsarcolemmal mitochondria; IFM, interfibrillar mitochondria.

### Mitochondrial Ca^2+^ uptake and Ca^2+^-induced swelling

**Figure 3:**
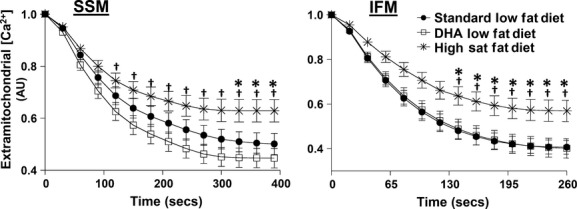
Ca^2+^ uptake in SSM and IFM. Data are presented as ± SEM. *P < 0.05 high saturated fat versus standard low fat, ^†^*P* < 0.05 high saturated fat versus DHA low-fat diet. The sample size was 8, 8, and 10 for SSM and 7, 9, and 9 for IFM for standard low fat, DHA, and high saturated fat, respectively.

**Figure 4:**
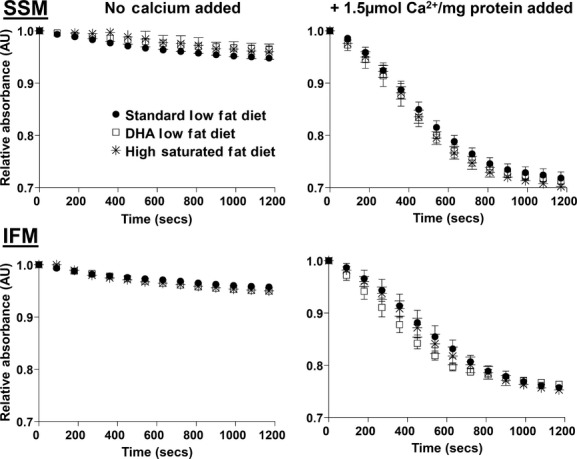
Ca^2+^-induced MPT measured in isolated mitochondria using a light scattering assay in SSM (top panels) and IFM (lower panels). The sample size was 10 for all groups.

Previous studies found that supplementation with DHA delayed MPT (O'shea et al. 2009; Khairallah et al. 2010b) and increased Ca^2+^ activation of pyruvate dehydrogenase in the mitochondrial matrix (Pepe et al. 1999), suggesting that it enhances the ability of mitochondria to take up Ca^2+^. Thus, we measured Ca^2+^ uptake by isolated mitochondria. There was a trend toward increasing the rate and total amount of Ca^2+^ taken up by the mitochondria with the DHA diet compared to the standard diet in SSM but not IFM, though this was not significant (Fig. 3). On the other hand, mitochondria from the rats on the high saturated fat diet had a significant decrease in the total amount of Ca^2+^ uptake compared to the other two diets for both IFM and SSM. No changes were seen in Ca^2+^-induced mitochondrial swelling at baseline or after the addition of Ca^2+^ (Fig. 4).

### Mitochondrial membrane microviscosity

**Figure 5:**
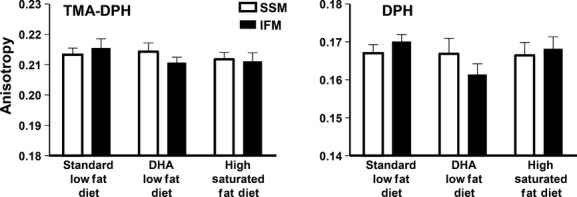
Mitochondrial membrane microviscosity measured using anisotropy of either TMA-DPH or DPH. The sample size was 8, 9, and 8 for both SSM and IFM for standard low fat, DHA, and high saturated fat, respectively.

Increasing the density of long-chain PUFAs through DHA supplementation has the potential to affect membrane fluidity. Thus we used fluorescence polarization to measure anisotropy, a measure of membrane microviscosity that is inversely related to membrane fluidity. No differences were seen among groups (Fig. 5).

## Discussion

This study compared the impact of two extreme diets, specifically a diet high in the very long-chain n3-PUFA DHA but low in total fat, and a high-fat diet rich in long-chain saturated fat, on mitochondrial function and phospholipid composition. The main finding is that despite dramatic differences in mitochondrial phospholipid side chain composition between the diets, there was surprisingly no difference in mitochondrial respiration, Ca^2+^-induced mitochondrial swelling, cardiac mass, LV chamber size, or systolic shortening. These findings illustrate the adaptability of cardiac mitochondria to extremes in dietary lipid composition and resultant changes in mitochondrial phospholipid fatty acid composition without major changes in cardiac function or structure.

Supplementation with DHA has previously been shown by our lab to readily incorporate into mitochondrial membranes and result in a proportional deletion in arachidonic acid (Khairallah et al. 2010a,b, 2012; Galvao et al. 2013). In this study, we saw a dramatic increase of almost threefold of DHA in mitochondrial membranes with ∼twofold decrease in arachidonic acid relative to the standard low-fat diet. In our previous studies with a similar level of DHA supplementation, we found delayed MPT in both normal and hypertrophied hearts rat hearts (Khairallah et al. 2010a,b, 2012) and in cardiomyopathic hamsters (Galvao et al. 2013). However, in contrast to our previous work (O'shea et al. 2009; Khairallah et al. 2010a,b, 2012; Galvao et al. 2013), we did not find evidence for an association between increased DHA and significant changes in Ca^2+^-induced MPT as assessed by the Ca^2+^-induced swelling assay, nor in the ability of isolate mitochondria to take up Ca^2+^. The reason for the lack of effect in the current investigation is unclear, but could be due the relatively short period of treatment (6 weeks compared to 10–26 weeks) (O'shea et al. 2009; Khairallah et al. 2010a,b, 2012; Galvao et al. 2013).

In addition to the effect on cardiac phospholipids, high intake of long-chain saturated fatty acids increased body and fat pad mass and elevated plasma free fatty acids and triglycerides, yet did not worsen LV function. High saturated fat intake had a relatively modest effect on mitochondrial phospholipid fatty acyl side chain composition, but significantly decreased mitochondrial Ca^2+^ uptake capacity but did not affect Ca^2+^-induced MPT, providing further evidence for a disconnect between phospholipid changes and mitochondrial function in healthy rats. Slower Ca^2+^ exchange between the mitochondrial matrix and the cytosol would likely result in a dampened Ca^2+^ activation of matrix dehydrogenases and slower activation of pyruvate oxidation and citric acid cycle flux in response to an increase in cardiac workload (McCormack et al. 1990; Zhou et al. 2007). The large increases in DHA in mitochondrial membranes and the dramatic increase in the DHA-precursor, DPA caused by the high saturated fat diet supports previous data in which saturated fat intake increases DHA (Shah et al. 2009; Galvao et al. 2012) which suggests that excess saturated fat may contribute to the elongation/desaturation of fatty acids to DHA. Increased DHA with high saturated fat intake may help explain observations from epidemiological data showing no adverse effect of saturated fat intake on incidence of cardiovascular disease (Astrup et al. 2011).

It is important to note that differences between SSM and IFM may impact the effects of the dietary interventions that we assessed. There is a slower rate of protein synthesis (Kasumov et al. 2013), and a greater respiratory capacity and resistance to MPT in IFM than SSM (Palmer et al. 1977, 1985, 1986; Hofer et al. 2009; Asemu et al. 2013). The present investigation was not designed to compare SSM and IFM, nevertheless there was clearly a higher yield of SSM than IFM (Table 3), which is consistent with recent studies by us in rats (Khairallah et al. 2010a,b, 2012) and mice (Papanicolaou et al. 2012). This is not a consistent finding, as classic work from the Hoppel laboratory (Palmer et al. 1977) and others (Heather et al. 2010) found ∼twofold greater yield of IFM than SSM using a similar isolation method. Other studies from this group (Riva et al. 2005; Rennison et al. 2007, 2008) and our laboratory (O'shea et al. 2009) observed no difference between IFM and SSM. It is possible that a higher yield of SSM than IFM is due to contamination with IFM in the isolation process, however, this is not possible to quantify as there is currently no established molecular marker of IFM.

An additional limitation of the present study is the incomplete assessment of mitochondrial coupling and proton leak. We observed no differences in the RCR and state 4 respiration rate as indicators of respiratory coupling, however, more sensitive methods over a range of inner mitochondrial membrane potentials were not employed (Brand and Nicholls 2011). Finally, it is important to note that the animals on the high saturated fat diet gained ∼40% more weight over the 8 weeks of treatment compared to the other two groups, which may have effected mitochondrial function. Future studies should include a weight matched food restricted group of animals to avoid this potentially confounding variable.

In conclusion, despite dramatic changes in mitochondrial phospholipid fatty acid side chain compositions and differential effects of mitochondrial Ca^2+^ uptake capacity, low-fat diets with and without DHA as well as a high saturated fat diet do not affect contractile function in healthy rats. Additionally, DHA levels in mitochondrial membrane phospholipids were increased with high saturated fat intake which may contribute to the beneficial effects of high saturated fat intake in some models of heart failure or pathological cardiac hypertrophy (Okere et al. 2005, 2006; Rennison et al. 2007, 2008; Sharma et al. 2007, 2008; Duda et al. 2008; Berthiaume et al. 2010; Galvao et al. 2012).
